# Singlet Oxygen Sensor Green is not a Suitable Probe for ^1^O_2_ in the Presence of Ionizing Radiation

**DOI:** 10.1038/s41598-019-44880-2

**Published:** 2019-06-10

**Authors:** Huanhuan Liu, Philippe J. H. Carter, Adrianus C. Laan, Rienk Eelkema, Antonia G. Denkova

**Affiliations:** 1 Department of Radiation Science and Technology, Delft University of Technology, Mekelweg 15, 2629 JB Delft, The Netherlands; 2 Department of Chemical Engineering, Delft University of Technology, van der Maasweg 9, 2629 HZ Delft, The Netherlands

**Keywords:** Fluorescent probes, Fluorescent dyes

## Abstract

A great number of fluorescent probes have been developed for detecting singlet oxygen (^1^O_2_), which is considered to be one of the most effective reactive oxygen species (ROS), especially in clinical applications. The commercially available fluorescent probe Singlet Oxygen Sensor Green (SOSG) is widely used due to its reported high selectivity to ^1^O_2_. In this study, we carried out systemic experiments to determine the activation of SOSG in the presence of ionizing radiation. The results show that the SOSG probe exhibits a pronounced fluorescence increase as a function of radiation dose delivered by gamma-rays as well as X-rays, in conditions where the formation of singlet oxygen is not expected. Furthermore, scavenger tests indicate that hydroxyl radicals may be involved directly or indirectly in the activation process of SOSG although the exact mechanism remains unknown.

## Introduction

Reactive oxygen species (ROS) occur naturally in human cells and are known to play a role in various cancerous processes^1^. At the same time, a number of cancer therapies rely on the generation of ROS to induce cell death^2^. Among all ROS, singlet oxygen (^1^O_2_) is considered to be the most effective in killing tumour cells and has been widely used in photodynamic therapy (PDT)^3^. To better understand the behaviour of photosensitizers generating ROS in PDT and consequently their biological effect, the detection of ROS, especially ^1^O_2_, is of great importance. However, the short lifetime and the low concentration of ^1^O_2_ tremendously complicate proper detection. Various approaches have been developed for the detection of ^1^O_2_, including direct measurement of the ^1^O_2_ luminescence at 1280 nm, electron spin resonance spectroscopy (ESR), fluorescent probes and others^4–6^. Fluorescent probes are widely employed due to their simplicity in utilization and high detection efficiency^7^. The commercially available probe Singlet Oxygen Sensor Green (SOSG) is currently the preferred choice for ^1^O_2_ detection due to its claimed specific sensitivity to singlet oxygen^8^.

**Figure 1:**
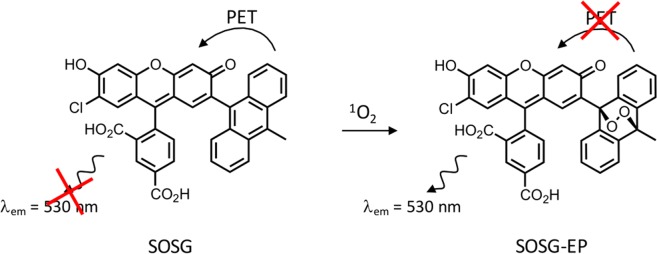
Chemical structure of SOSG and the formation of SOSG-EP upon interaction with ^1^O_2_, leading to activation of fluorescence output.

As shown in Fig. 1, the SOSG molecule has two parts: a trapping moiety and a fluorophore^9^. The anthracene-derived trapping moiety, being an electron donor, quenches the luminescence of the fluorophore by photo-induced electron transfer. In an environment containing ^1^O_2_, the trapping moiety will react with ^1^O_2_ and form an endoperoxide anthracene moiety, which has a lower energy for the highest occupied molecular orbital (HOMO) than that of the fluorophore. Removing the quenching ability of the anthracene leads to fluorescence (FL) emission of the fluorophore under light excitation with a peak at around 530 nm. Despite the many advantages of SOSG, it was previously shown that this probe is not entirely reliable under certain conditions. The main drawback of SOSG is that, for instance when irradiated with UV light, its endoperoxide derivative acts as a photosensitizer itself, generating singlet oxygen which then induces even more fluorescence emission of SOSG^9–11^. Moreover, photo-bleaching of SOSG is observed even for short light exposure times^9^.

In photodynamic therapy, singlet oxygen is typically generated by light irradiation of a photosensitizer. Due to absorption and scattering effects of tissue, the penetration depth of light in living tissue can be very shallow, which limits further clinical application^12^. To overcome this challenge, one of the promising options is to combine PDT with radiotherapy. For example, nano scintillators conjugated to photosensitizers were developed which can convert ionizing radiation to light, producing ROS^13–15^. Very recently it was also suggested that radioactive isotopes (e.g. ^18^F, ^64^Cu) producing so-called Cerenkov light could function as internal light sources, which in combination with photosensitizers can be applied to induce tumour cell death^3,16^. Moreover, in radiation-involved therapies, the generation of ROS is also an indirect strategy to reduce the growth of tumour cells. In these therapies, probes are also used to prove the generation of ^1^O_2_^17–19^.

As far as we know, the influence of ionizing radiation on the photochemical behaviour of such probes has not been comprehensively evaluated. In this paper, the photochemical performance of SOSG was assessed when exposed to ionizing radiation under conditions similar to other studies^13,19^. In this study we used gamma-rays (γ-rays) and X-rays, which are common types of ionizing radiation in external radiotherapy. After irradiation, the UV-vis absorption and fluorescence emission of SOSG were determined. We found that, under conditions that are unlikely to generate ^1^O_2_, the SOSG probe shows an increasing fluorescence emission as the radiation dose is increased, without any detectable changes in UV-vis spectrum. Furthermore, scavenger tests suggest that the generation of hydroxyl radicals may be related to the increase in fluorescence emission of SOSG.

## Results and Discussion

**Figure 2:**
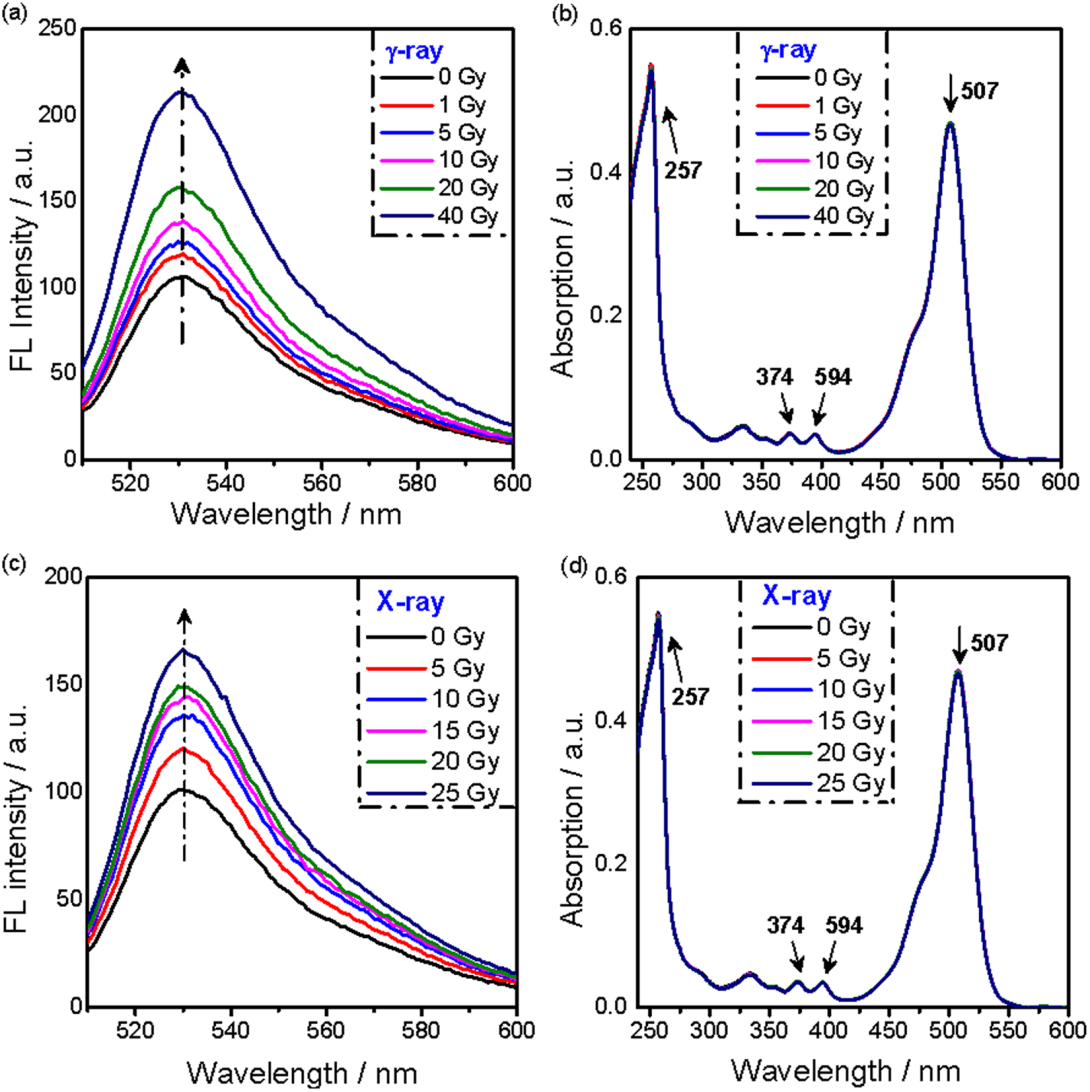
(**a**) Fluorescence spectra of SOSG solutions (5 µM) for varied radiation doses delivered by a ^60^Co source (λ_ex_ = 504 nm); (**b**) UV-vis absorption spectra of SOSG solutions (5 µM) for different radiation doses delivered by a ^60^Co source; (**c**) Fluorescence spectra of SOSG solutions (5 µM) for different X-ray doses; (**d**) UV-vis absorption spectra of the SOSG solution (5 µM) for different X-ray doses.

The effect of ionizing radiation on the photochemical properties of SOSG was studied systematically using two different external radiation sources, i.e., a cobalt-60 (^60^Co) γ-ray source and an X-ray source. The fluorescence spectra of SOSG after exposure to gamma rays are presented in Fig. 2a, showing that the FL intensity of the SOSG solution increased with increasing radiation doses. Figure 2b shows the UV-vis absorption spectra of SOSG at the same irradiation conditions, where the maximum band at 507 nm belongs to the fluorescein moiety, while the large peak at 257 nm and the two smaller peaks at 374 and 394 nm correspond to the methylanthracene moiety^9^. No noticeable changes were observed in the UV-vis spectra as the radiation dose increases, suggesting that the structure of SOSG is not affected or the concentration of activated SOSG remains low. To check whether SOSG also reacts with ionizing photons of lower energy, we exposed the probe solutions to X-rays with a maximum energy of 320 keV and monitored the fluorescence spectra and UV-vis absorption at different doses. As shown in Fig. 2c, the X-ray exposure also induces fluorescence emission of the SOSG probe, and the FL intensity exhibits an upward trend with increasing radiation doses. In fact, the FL intensity increase is comparable to the raise observed when using the ^60^Co source for the same radiation dose, showing that this process is most likely not dependent on the photon energy. The UV spectra of SOSG appear unchanged for all X-ray radiation doses (Fig. 2d).

As a check we also exposed the SOSG samples to UV light sources using two different wavelengths (365 nm and 400 nm). These experiments show that the SOSG fluorescence intensity increases significantly as function of irradiation time (Fig. S1), which is likely caused by the generation of ^1^O_2_ in the presence of UV light. Meanwhile, in UV-vis the intensity of the peaks at 257, 507 and 394 nm decreases upon UV irradiation, caused by the formation of SOSG-EP^10^.

Ionizing radiation can have direct and indirect effects on the behavior of SOSG. In direct interaction, ionizing radiation can result in breaking of chemical bonds, changing the SOSG structure. Indirect effects result from the generation of reactive oxygen species in the aqueous solution which can react with SOSG, or influence the intermolecular electron transfer processes, resulting in the fluorescence emission of SOSG. According to the unchanged UV-vis spectra of the irradiated SOSG samples, chemical bonds are most likely not broken which is expected considering the nature of radiation (i.e. photons) and the low radiation dose (max 25 Gy). Therefore, we focused mostly on any possible indirect effects.

**Figure 3:**
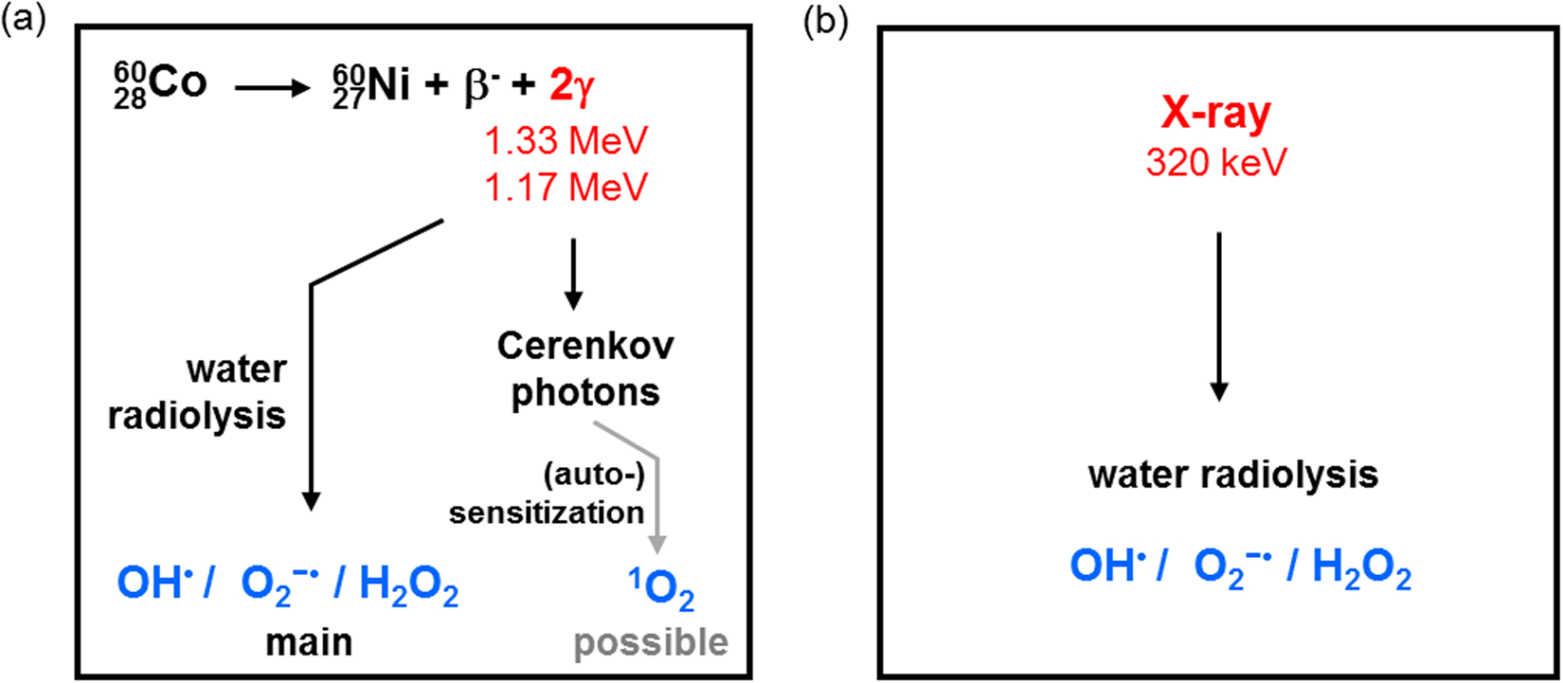
Possible reactive oxygen species generated under (**a**) gamma ray radiation and (**b**) X-ray (320 keV) radiation of aqueous SOSG solutions.

Figure 3 shows various photochemical processes that may take place in the aqueous SOSG solution induced by ionizing radiation, which may influence the photochemical performance of the SOSG probe. The ^60^Co is a radioisotope that decays by beta minus emission, producing in the process two energetic γ-rays of 1.17 MeV and 1.33 MeV^20^. These photons can interact with water via the Compton effect, giving part of their energy to electrons. The energy of these photons is high enough to provide electrons with energies above the threshold for the production of Cerenkov radiation (261 keV in water)^21^. It should be mentioned that Cerenkov light has a wide spectrum ranging from 250 to 800 nm with a maximum around 360 nm^22,23^, which overlaps with the absorption range of SOSG resulting in some probability of ^1^O_2_ generation. Ionizing radiation also leads to the radiolysis of water which produces various reactive oxygen species, including OH^•^, O_2_^−•^ and H_2_O_2_^24^. This means that both processes, i.e., the radiolysis of water and the Cerenkov induced singlet oxygen production by SOSG, can in theory generate ROS (Fig. 3a). Similar to γ-rays, X-rays could also trigger the radiolysis of water, leading to a complex mixture of different reactive oxygen species. The X-rays that we used in this study have a maximum energy of 320 keV, however, the energy that could be transferred to the Compton electron is less than 180 keV, which is not sufficient to induce Cerenkov light (Fig. 3b).

**Figure 4:**
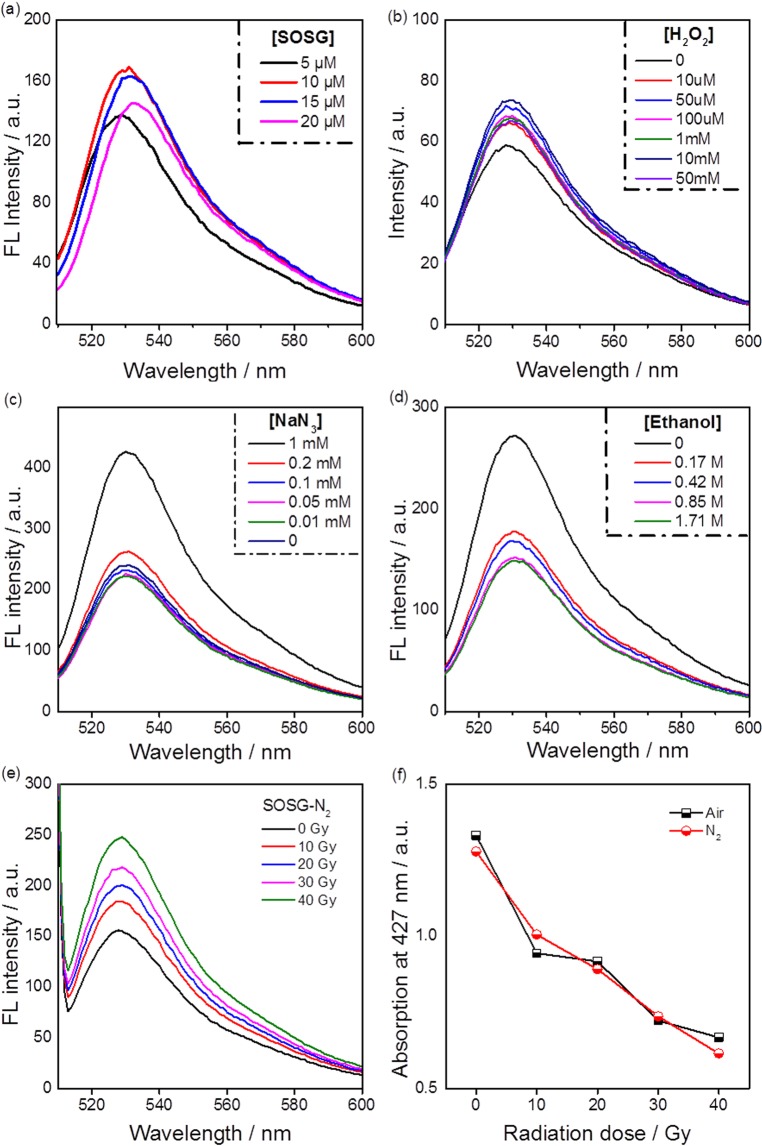
Fluorescence spectra (λ_ex_ = 504 nm) of SOSG solutions (**a**) with varied SOSG concentrations, (**b**) reacted with H_2_O_2_ solutions with varied concentrations, (**c**) reacted with NaN_3_ solutions with different concentrations and (**d**) in the addition of varied amount of ethanol under 20 Gy radiation dose from a ^60^Co source; Fluorescence spectra of SOSG solutions (5 µM) (**e**) saturated with N_2_ for varied radiation doses delivered by a ^60^Co source; (**e**) The FL intensity determined at 529 nm of air-saturated and N_2_-saturated SOSG solutions as function of radiation dose.

In order to determine what causes the increase in FL emission of SOSG when using ionizing radiation, we first measured the effect of SOSG concentrations on the fluorescence performance. As shown in Fig. 4a, there is no clear trend of the FL intensity when the concentration increases, implying the observed FL emission at a fixed radiation dose does not depend on [SOSG]. In another words, the radiation induced process in the solvent dominates the FL emission. Consequently, the influence of various reactive oxygen species on the SOSG fluorescence was measured. The addition of H_2_O_2_, a product of water radiolysis, to the SOSG solution was found to lead to only a very slight increase in FL intensity at H_2_O_2_ concentrations much higher than those expected to be formed for this radiation dose (the H_2_O_2_ generation caused by radiolysis of water can be found in Fig. S3). Considering the increasing FL intensity of SOSG probe under γ-ray and X-ray irradiation, H_2_O_2_ is unlikely to be the species responsible for the FL phenomenon.

Subsequently, we performed scavenger tests involving different ROS scavengers. First, we used NaN_3_, an effective scavenger of ^1^O_2_^11^, to check the effect of the possible singlet oxygen formation. According to the results shown in Fig. 4(c), the FL intensity presents a negligible decrease at lower NaN_3_ concentrations (i.e., 0.01, 0.05 and 0.1 mM), and then significantly increases when the concentration of NaN_3_ increases to 0.2 mM and 1 mM. The low [NaN_3_] experiments suggest a negligible contribution of singlet oxygen. The large FL intensity increase at high [NaN_3_] may be ascribed to the interaction between SOSG and N_3_^•^ radicals, a strong oxidant species generated by the reaction between N_3_^−^ and OH^•^^25^. A large amount of hydroxyl radicals are formed under γ-ray radiation as shown in Fig. S4. Therefore, NaN_3_ is not considered to be a proper scavenger of ^1^O_2_ for this study due the presence of hydroxyl radicals. In contrast, the introduction of small amounts of ethanol, a typical scavenger of hydroxyl radical (OH^•^)^26^, leads to an evidently lower FL signal. Moreover, the FL signal exhibits a declining trend with the increasing addition of ethanol. Other common scavengers of OH^•^, i.e., NaI and methanol, also exhibit inhibition of the FL intensity of SOSG (Figs S6, 7). The suppression of hydroxyl radicals during water radiolysis will also affect the formation of other radicals, which might interact with SOSG^27^. Therefore, it is not possible to state with certainty that the presence of OH^•^ is the sole cause of the observed increase in FL intensity.

Since NaN_3_ could not serve as a suitable scavenger of ^1^O_2_, we carried out an indirect approach to evaluate the role of ^1^O_2_ in these experiments by saturating the solutions with N_2._ In N_2_ saturated solutions, ^1^O_2_ formation is impossible. As shown in Fig. 4(e), the fluorescence of SOSG in a N_2_-saturated aqueous solution shows an increasing signal as function of radiation dose. Furthermore, comparing the FL intensity of SOSG solutions saturated with N_2_ or normal air atmosphere (Fig. S8) shows no significant difference, which strongly suggests that the fluorescence of SOSG in these experiments is not induced by singlet oxygen. Although γ-ray radiation delivered by ^60^Co is able to generate Cerenkov light in the UV-Vis range, this light is likely not enough to activate the SOSG probe, in contrast to much higher intensity UV sources (365 nm, 400 nm) that were used in our experiments.

In addition to the SOSG probe, we also checked the photochemical behavior of another two probes, i.e., DPBF (1,3-diphenylisobenzofuran) and ABDA (9,10-antherachenediyl-bis(methylene) dimalonic acid), which are widely used to detect the formation of ^1^O_2_^17,28–31^. As illustrated in Figs S9, 10, both probes were activated in the presence of ionizing radiation. Comparison of the photochemical performance under different atmospheres (Figs S9b and S10b) again indicates that the activation processes are not caused by the formation of ^1^O_2_.

**Figure 5:**
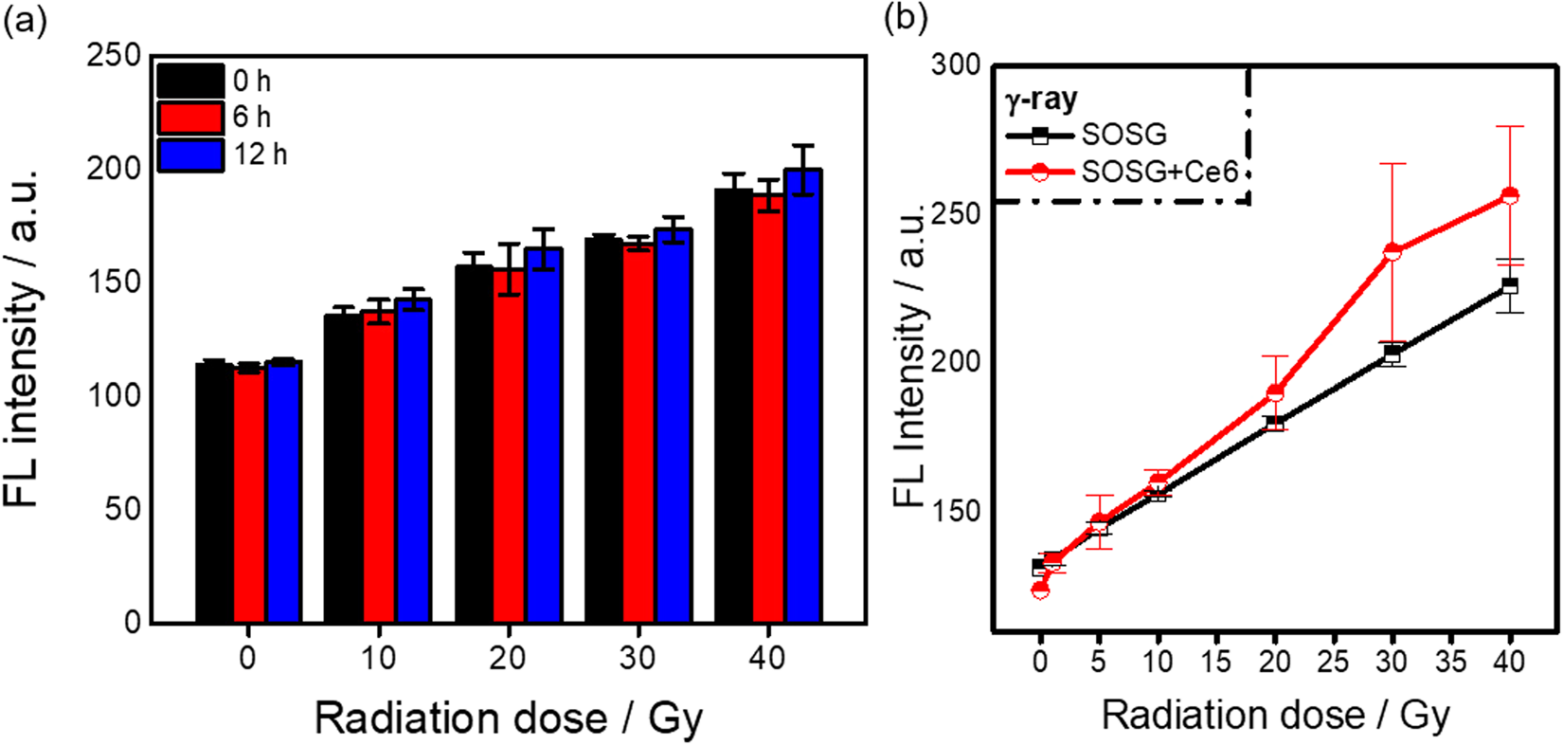
(**a**) Comparison of the fluorescence intensity at 529 nm for SOSG solutions (5 µM) at different time intervals after γ-ray irradiation of different radiation doses; (**b**) Comparison of the fluorescence intensity at 529 nm between pure SOSG solutions (5 µM) and SOSG solutions (5 µM) containing Ce6 (5 µM) (λ_ex_ = 504 nm). (Error bars represent the standard deviation of at least 3 replicates).

In order to determine the long-term influence of ionizing radiation on the photochemical performance, we measured the fluorescence of SOSG at different time intervals after exposure to various radiation doses. Figure 5(a) clearly indicates that the induced fluorescence is permanent which in turn implies that the molecular structure of SOSG might be affected. However, the measured mass spectra (Fig. S2) show no noticeable change in molecular weight after exposure to ionizing radiation, when compared to original SOSG solutions. It is, nevertheless, possible that the amounts of SOSG that were affected were so small that they could not be properly detected, which hinders establishment of the mechanism leading to the observed phenomenon.

Additionally, since Cerenkov light is studied as a promising internal light source for photodynamic therapy^3,20^, we decided to study the activation of SOSG in the presence of the common photosensitizer chlorin-e6 (Ce6), which has been proven to be excited by exposure to Cerenkov light^16^. Ce6 is a typical photosensitizer that is known to be highly efficient in the generation of singlet oxygen and is widely used in photodynamic therapy. We exposed a simple SOSG solution and a SOSG solution mixed with Ce6 to gamma rays originating from the ^60^Co source. Figure 5(b) shows that the FL intensity at 529 nm increases as a function of the gamma dose for both solutions. The FL values in the presence of Ce6 are somewhat higher than those in the pure SOSG solution at higher doses, although no firm conclusion can be drawn due to the large uncertainty of the measured FL intensity. Still, it is possible that at higher radiation doses Ce6 generates singlet oxygen through the Cerenkov effect. As a check we also exposed the same solutions to UV light sources of two different wavelengths (365 nm and 400 nm), showing that the SOSG fluorescence intensity increases significantly in the presence of Ce6 (Fig. S11). Although there is some increase of FL intensity observed from the pure SOSG solution induced by the UV light sources, it is much smaller than the signal from the solution containing the photosensitizer. These results also support that the SOSG probe is very sensitive to singlet oxygen, under common experimental conditions.

## Conclusions

In this paper, we studied the effect of ionizing radiation on the photochemical behavior of SOSG, showing that this probe becomes fluorescent when exposed to either γ-rays or X-rays. SOSG shows increased fluorescence intensity as function of radiation dose, which appears not to be related to singlet oxygen formation. Scavenger tests reveal that the suppression of hydroxyl radicals lead to a decrease in induced fluorescence intensity, which suggests that these species play some role in the activation of the SOSG probe in the presence of ionizing radiation. In contrast, when exposed to UV-light sources, SOSG is efficiently activated through the formation of the SOSG endoperoxide, caused by reaction with singlet oxygen. In addition, another two commercially available probes used for the detection of singlet oxygen, i.e. 1,3-diphenylisobenzofuran and 9,10-antherachenediyl-bis(methylene) dimalonic acid, were also evaluated under ionizing radiation conditions. These two probes appeared also to be activated by ionizing radiation. The exact mechanism leading to the activation of SOSG as well as the two other probes when exposed to ionizing radiation remains unclear but this study does clearly demonstrate that such probes should be cautiously used under such conditions.

## Experimental Section

### Materials

Singlet Oxygen Sensor Green was purchased from Thermo Fisher; Chlorin-e6 (C_34_H_36_N_4_O_6_) was purchased from Bio-connect life sciences (Huissen, the Netherlands); Sodium azide, sodium acetate, ammonium molybdate ((NH_4_)_6_Mo_7_O_24_•4H_2_O), potassium iodide (KI), sodium iodide (NaI) were obtained from Merck (Darmstadt, Germany); Methanol, hydrogen peroxide (H_2_O_2_, 30% (W/W)), acetic acid, 1,3-diphenylisobenzofuran (DPBF) and 9,10-antherachenediyl-bis(methylene) dimalonic acid (ABDA) were bought from Sigma Aldrich (Zwijndrecht, the Netherlands). Ethanol was bought from Brenntag (Zwijndrecht, The Netherlands). Dimethyl Sulfoxide (DMSO) was bought from Biosolve B. V. (Valkenswaard, The Netherlands). All chemicals were used without further treatment. Water used in these experiments was prepared with the in-house Milli-Q system from Merck Millipore.

### Sample preparation

All samples were prepared in dim environment.

SOSG stock solution (10 µM): Typically, 100 µg of SOSG was dissolved in 33 µL of methanol, and then this yellow solution was added to 16.467 mL of MQ water. For preparing SOSG solutions with different concentrations, the volume of MQ water was changed as required. ABDA stock solution (2 mM): 1.64 mg of 9,10-anthracenediyl bis(methylene) dimalonic acid was dissolved in 2 mL of DMSO, then ultrasonicated for 10 min. DPBF stock solution (2 mM): 2.7 mg of 1,3-diphenylisobenzofuran was dissolved in 5 mL of DMSO and ultrasonicated for 10 min. Ce6 stock solution (10 µM): 1.79 mg of Ce6 was dissolved in 300 mL MQ water and ultrasonicated for 20 min to obtain a Ce6 solution. NaN_3_ stock solution (21 mM): 6.825 mg of NaN_3_ was dissolved in 5 mL of MQ water and ultrasonicated it for 10 min to obtain a transparent solution. NaN_3_ solutions with the concentration of 4.2, 2.1, 1.05 and 0.21 mM were obtained by dilution of the NaN_3_ stock. KI stock solutions (1 M): 2.656 g of KI was added to 16 mL of MQ water and ultrasonicated for 10 min. Ammonium molybdate ((NH_4_)_6_Mo_7_O_24_•4H_2_O) stock solution (5 mM): 98.9 mg of (NH_4_)_6_Mo_7_O_24_•4H_2_O was dissolved in 16 mL of CH_3_COONa/CH_3_COOH buffer solution (1M) then ultrasonicated for 10 min.

SOSG sample (5 µM): 1 mL of SOSG solution was added to a glass vial (4 mL) wrapped tightly with aluminium foil, followed by the addition of 1 mL MQ water. A SOSG (5 µM) + Ce6 (5 µM) solution: 1 mL of SOSG stock solution was added to a glass vial which was also tightly wrapped with aluminium foil, followed by the addition of 1 mL of Ce6 stock solution. DPBF sample: 0.1 mL of DPBF base was diluted by 2 mL of MQ water, achieving a final concentration of 0.1 mM. ABDA samples: 0.05 mL of ABDA base and 2 mL of MQ water was mixed well to form a mixture with the concentration of 0.05 mM.

Samples for the scavenger tests: NaN_3_ addition: 0.1 mL of NaN_3_ solution with different concentrations were add to 2 mL of SOSG sample in a closed covered glass vials; Ethanol addition: expected amounts of ethanol were added to the SOSG samples (5 µM). Before radiation, all samples were thoroughly mixed.

N_2_-saturated solutions were prepared by bubbling the samples with N_2_ for 20 min.

### Irradiation experiments

Gamma irradiation: A Cobalt 60 (^60^Co) radioactive source (GC220, Nordion) was used for the gamma irradiations. The aluminium foil covered vials were placed in the centre of the source. The exposure periods were controlled to obtain irradiation doses of 1, 5, 10, 20 and 40 Gy; (The dose rates of ^60^Co was calculated using Fricke dosimetry corrected for the 2,778 day half-life of Cobalt-60).

X-ray irradiation: The X-ray irradiation was carried out using an X-ray source (Philips MCN 321 variable-energy X-ray tube) with a voltage of 320 kV and current of 3 mA, without filter. The samples were placed on a horizontal platform located 50 cm from the X-ray window (the dose rate was 1.36 Gy/min). The exposure doses were 5, 10, 15, 20 and 25 Gy.

UV (365 nm/254 nm) irradiation: A commercial UV source (UVGL-58 handheld UV Lamp) was employed as the light source. The aluminium foil was first removed from the vials, followed by placing these vials in a dim box. Then the light source was turned on to irradiate these samples for fixed periods of time.

UV (400 nm) irradiation: A LED light connected to the FL spectrometer was employed as the light source. Samples were firstly transferred from vials to cuvettes, and then these cuvettes were placed in the sample tank, the light source was turned on (0.3~0.5 A and 0.3 kV) to start the exposure.

### Characterization

Mass spectra of SOSG solutions were recorded using an ESI mass spectrometer (LCMS-2010A, Shimadzu). A UV-vis-NIR spectrophotometer (UV-6300PC, VWR) was used to measure the optical absorption of the prepared samples. A Cary Eclipse Fluorescence Spectrophotometer (Agilent technologies) was employed to characterize the fluorescence emission spectra of the samples. The emission peak for SOSG was located at 529 nm using a 504 nm excitation light. The slits of excitation and emission are 5 nm, if not be mentioned specially.

The generation of H_2_O_2_ was detected by Ghormley’s triiodide method^32^: the standard samples were prepared by diluting the H_2_O_2_ to obtain different concentrations; then 0.1 mL of the KI solution and 0.1 mL of the ammonium molybdate solution was added, and leaving it to react for 10 min, after which the UV intensity at 350 nm was measured^33^.

Detection of H_2_O_2_ generation after ionizing radiation exposure: after the radiation treatment, 0.1 mL of the KI solution and 0.1 mL of the ammonium molybdate stock solution was immediately added to the samples, and the UV spectra was measured after reaction time of 10 min.

## Supplementary information

Supplementary information
