# Supplementary material for: Singlet Oxygen Sensor Green is not a Suitable Probe for 1O2 in the Presence of Ionizing Radiation
Source: Sci Rep. 2019 Jun 10;9:8393. doi: 10.1038/s41598-019-44880-2 (PMC6557857; doi:10.1038/s41598-019-44880-2)
Supplement: Supplementary file 1 — Supplementary information [file 41598_2019_44880_MOESM1_ESM.docx]

Supplementary Information

Singlet Oxygen Sensor Green is not a Suitable Probe for ^1^O_2_ in the Presence of Ionizing Radiation

Huanhuan Liu^1,2^, Philippe J. H. Carter^1^, Adrianus C. Laan^1^, Rienk Eelkema^*2^, Antonia G. Denkova^*1^

**Supplementary Figures**


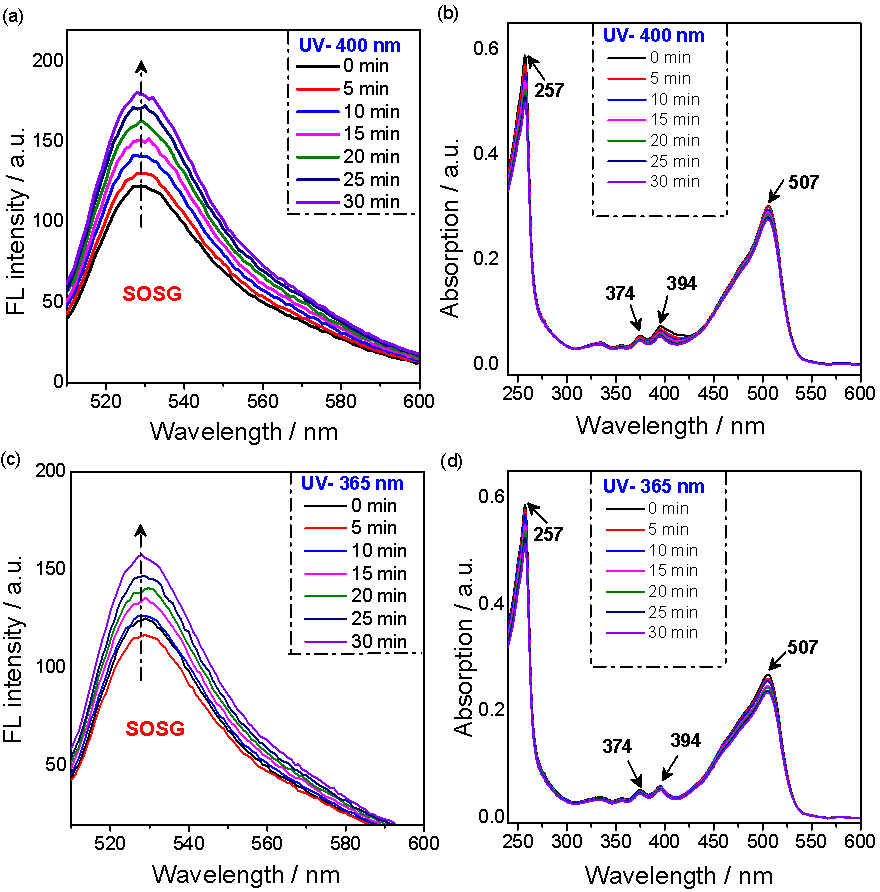


Figure S1. (a)Fluorescence spectra (λ_ex_= 504 nm) and (b) UV-vis spectra of SOSG solutions (5 µM) after irradiation with UV light at 400 nm; (c)Fluorescence spectra and (d) UV-vis spectra of SOSG solutions (5 µM) after irradiation with UV light at 365 nm.

The fluorescence emission in Figure S1(a,b) is predominantly due to the generation of singlet oxygen. In contrast to the UV-vis absorption spectra of SOSG irradiated by γ- and X-ray sources, the spectra of UV irradiated SOSG show noticeable decreasing absorption peaks (257, 365 nm and 400 nm). This effect is caused by endoperoxide (SOSG-EP) formation.^1^ Meanwhile, the intensity of the 507 nm peak also shows a decreasing trend, which may be due to the photo bleaching of SOSG.


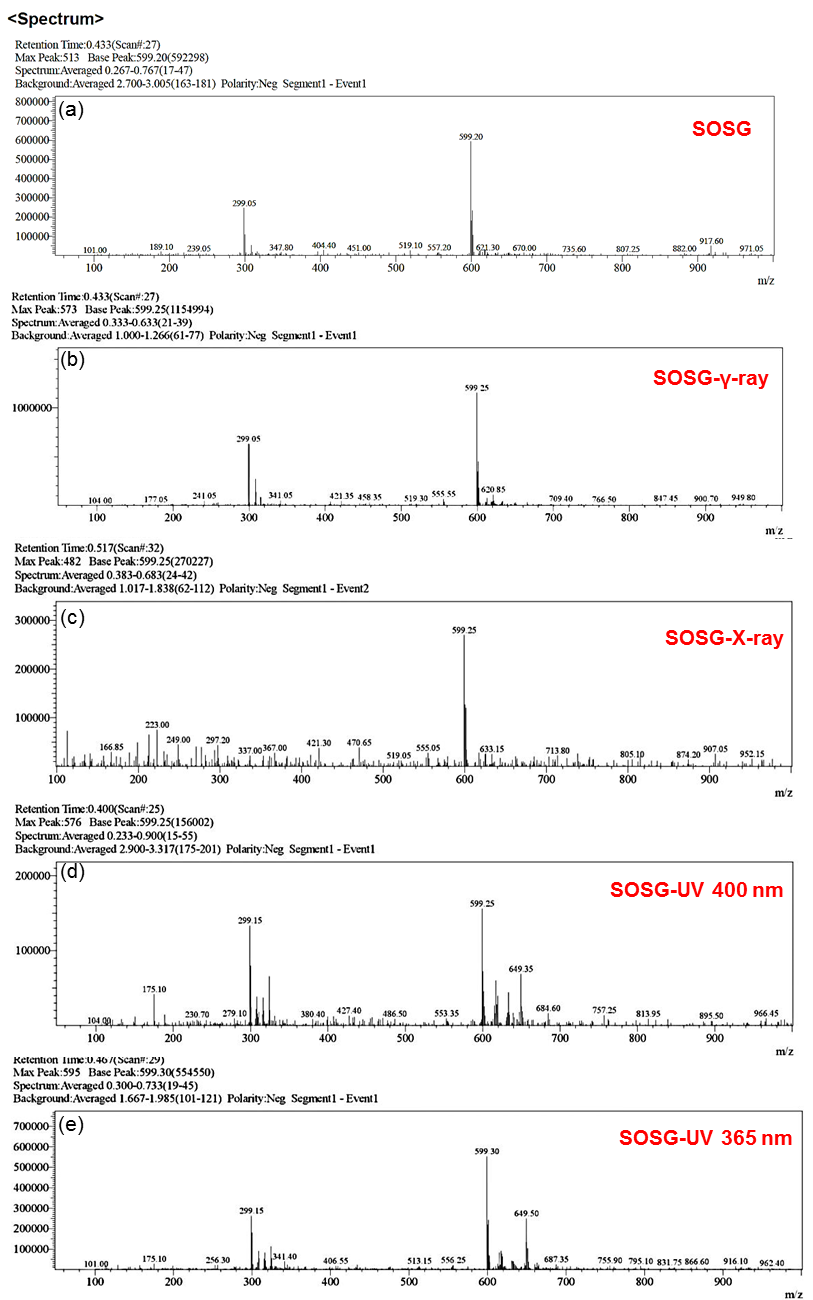


Figure S2. ESI Mass spectrum (negative mode) of (a) a SOSG solution; (b) a SOSG solution exposed to γ-ray radiation dose of 40 Gy; (c) a SOSG solution exposed to X-ray radiation dose of 20 Gy; (d) a SOSG solution exposed to 400 nm UV light for 50 min; (e) SOSG exposed to 365 nm UV light for 50 min. (concentration of the SOSG solution is 25 µM).

The mass spectra (ESI-MS) in Figure S2 show differences depending on the irradiation conditions. The spectra of SOSG solutions exposed to X- and γ-ray remain similar to that of the non-irradiated SOSG solution with a major peaks at m/z=599 and m/z=299, which correspond to the molecular structure of SOSG (mono-anion and di-anion respectively); spectra of SOSG exposed to UV sources (Figure S2(d,e)) exhibit a clear peak located at m/z=649, associated to the water adduct of SOSG-EP.^1^

Figure S3. (a) shows the standard curve obtained through Ghormley’s triiodide method, which achieves a linear relationship between UV intensity (350 nm) and H_2_O_2_ concentrations. Figure S3(b,c) show that the H_2_O_2_ concentration in aqueous solutions exposed to γ-ray radiation increases as function of radiation dose. According to the calibration line, the generation of H_2_O_2_ under 40 Gy is ~25.3 µM. Figure S3(d) demonstrates that the generation of H_2_O_2_ in pure water and in the SOSG solution is highly dependent on the radiation dose.


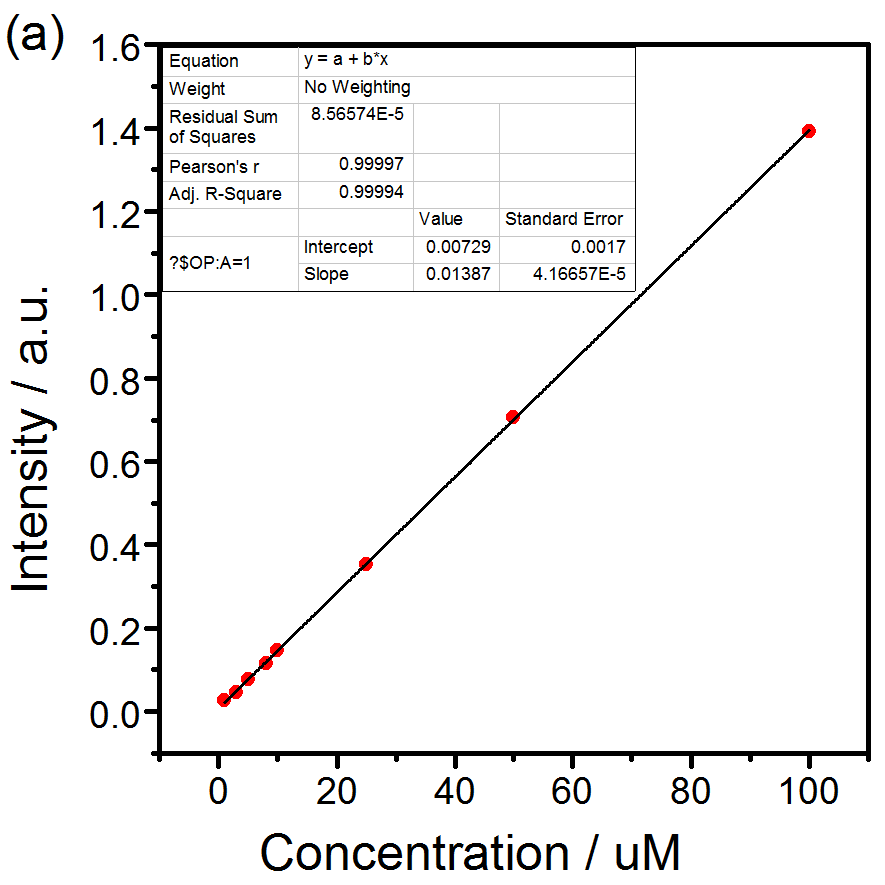

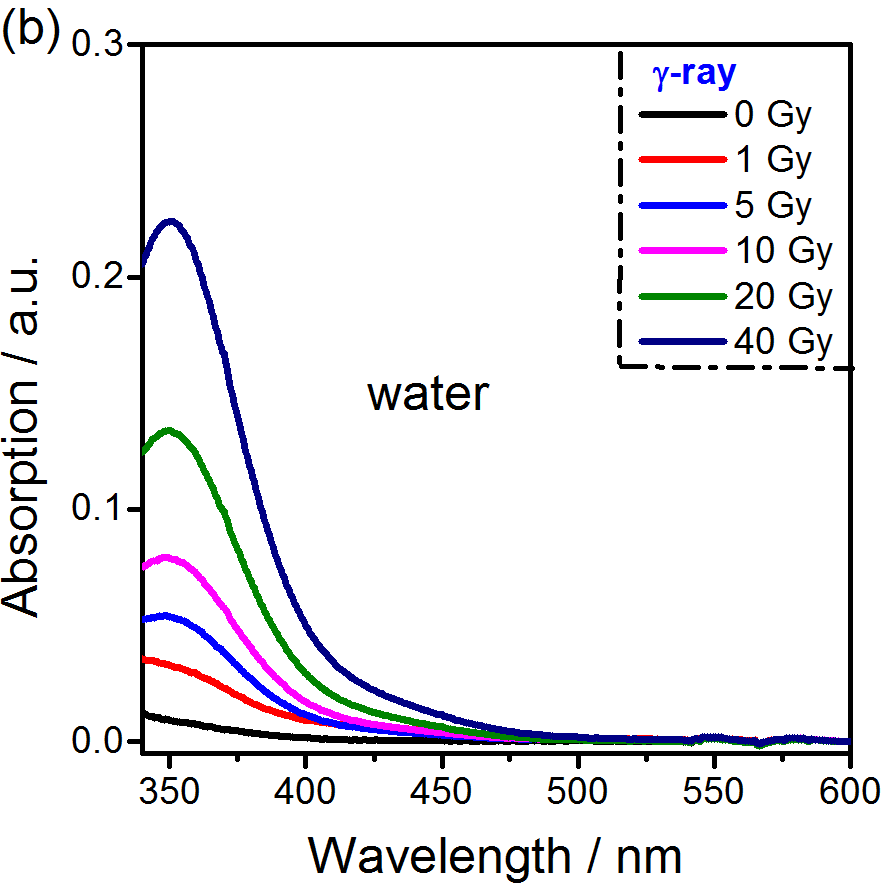


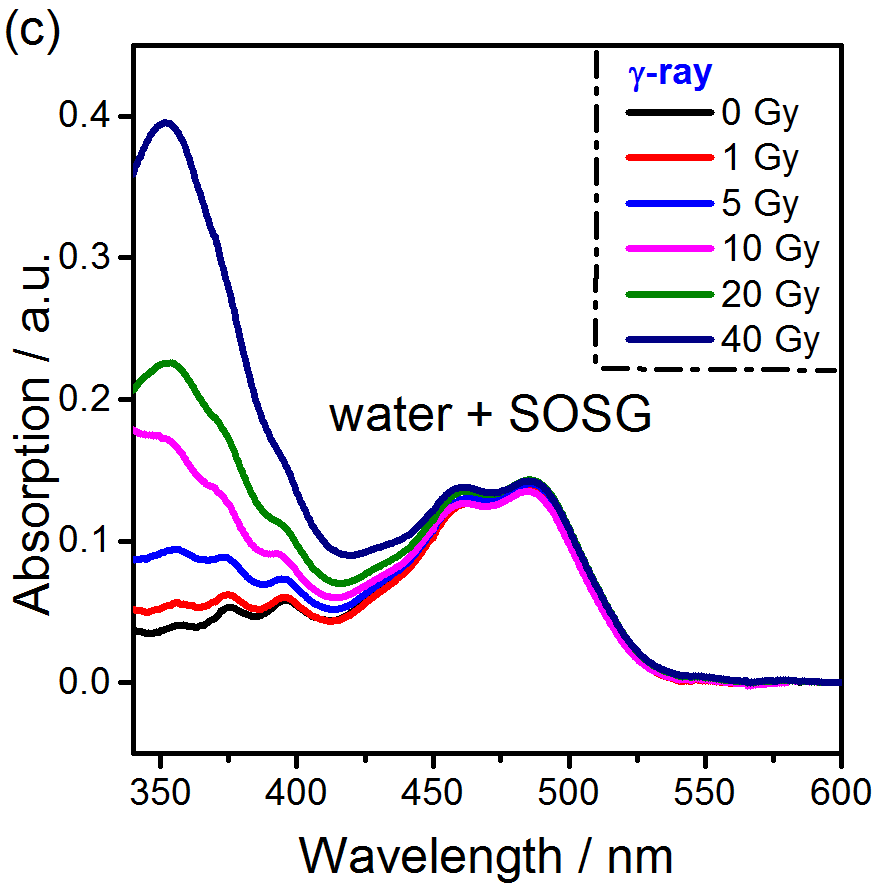

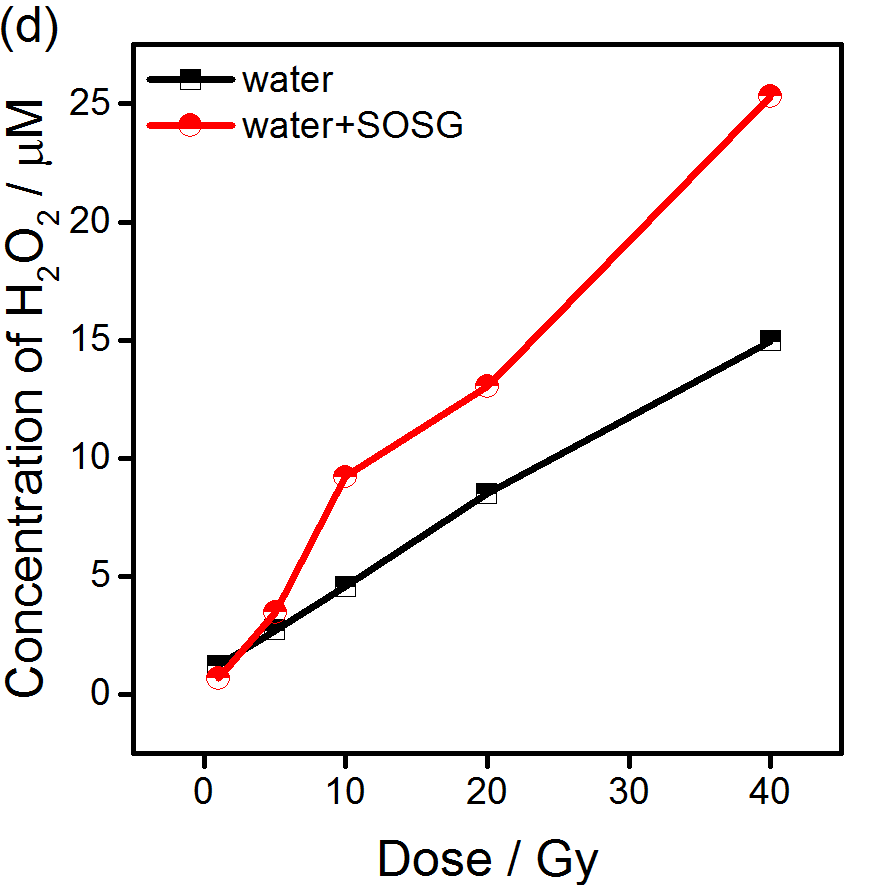


Figure S3. (a) Standard curve of H_2_O_2_ obtained through Ghormley’s triiodide method; the UV spectrum for radiation exposed (b) water and (c) the SOSG solution (5 µM) by addition of KI and ADM solution; (d) The comparison of H_2_O_2_ generation in water and the SOSG solution (5 µM). (For detection of H_2_O_2_ concentration, the KI solutions and the ammonium molybdate stock solutions were added to water and the SOSG solutions used.)

APF (Aminophenyl fluorescein) is a typical probe for the detection of hydroxyl radical. Figure S4 shows that even at a low dose, i.e., 1 Gy, γ-ray irradiation of aqueous solutions can induce the activation of this probe, indicating the generation of hydroxyl radicals.


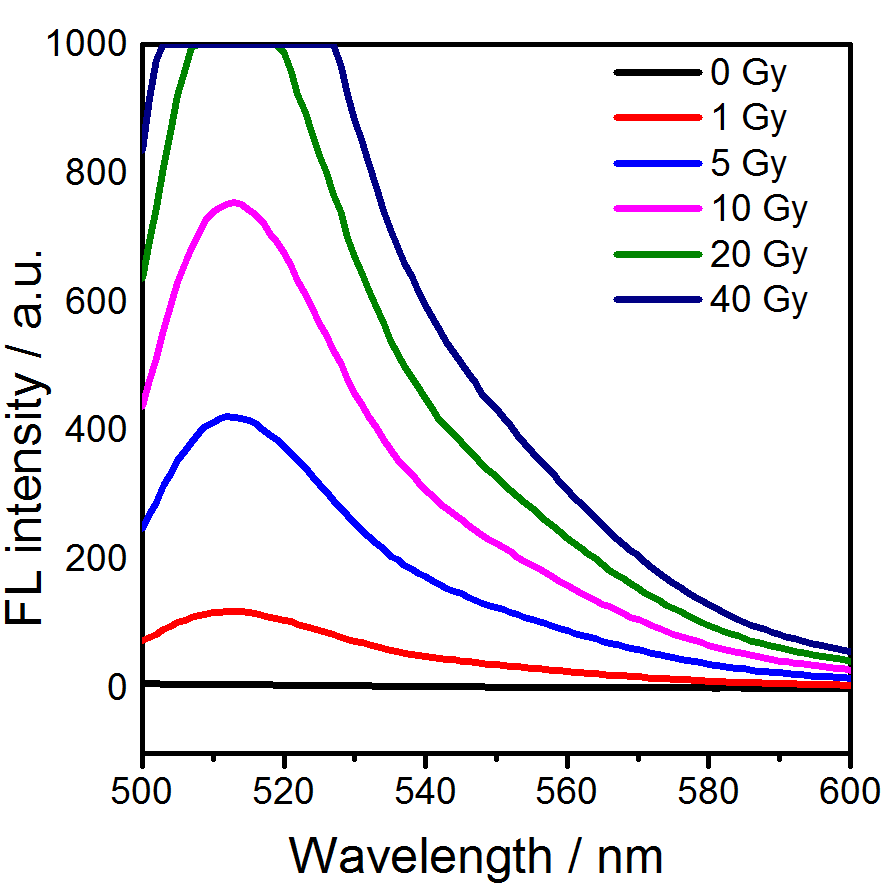


Figure S4. Fluorescence spectra (λ_ex_= 480nm) of a 5µM APF aqueous solution as a function radiation dose delivered by ^60^Co source.

Figure S5 shows the UV-vis spectra of SOSG after irradiation experiments and the addition of various ROS scavengers. According to Figure S5(a), no structural changes were observed in SOSG molecules when exposed to 40 Gy of γ-ray radiation, in the presence or absence of EtOH. The introduction of NaN_3_ also did not affect the structure of the SOSG molecule (Figure S5(b)). However, when exposed to radiation the mixture of NaN_3_ and SOSG exhibited an evident decrease of the absorption at 507 nm which indicates a change of the fluorescein moiety, possibly due to reaction with N_3_^•^ radicals.


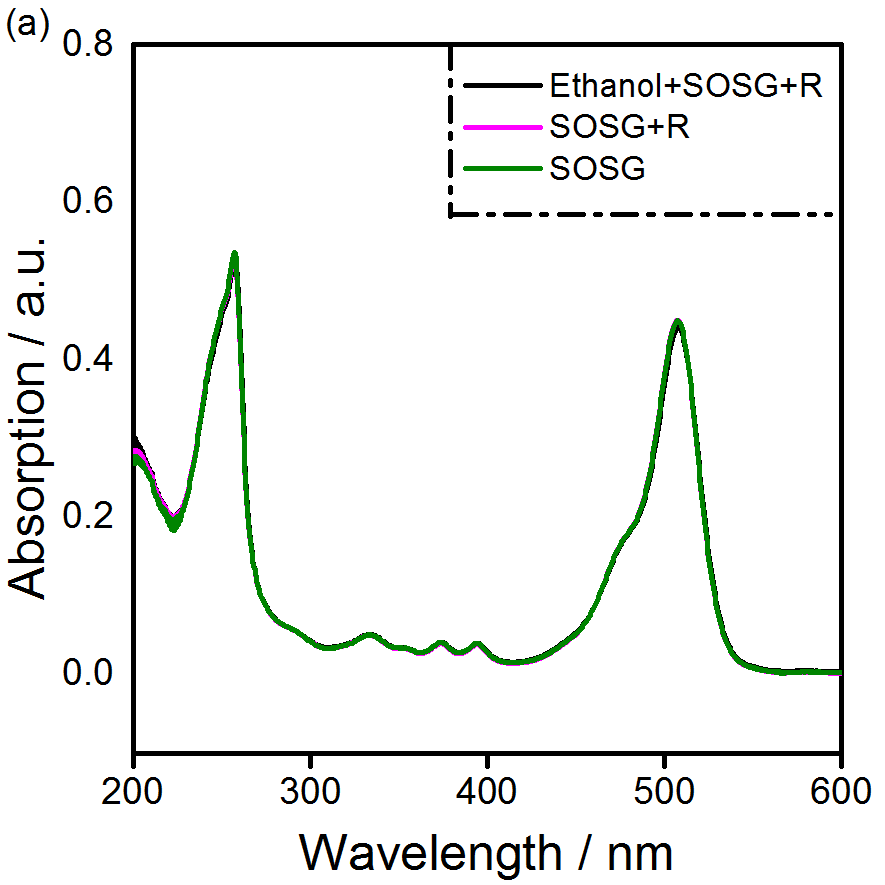

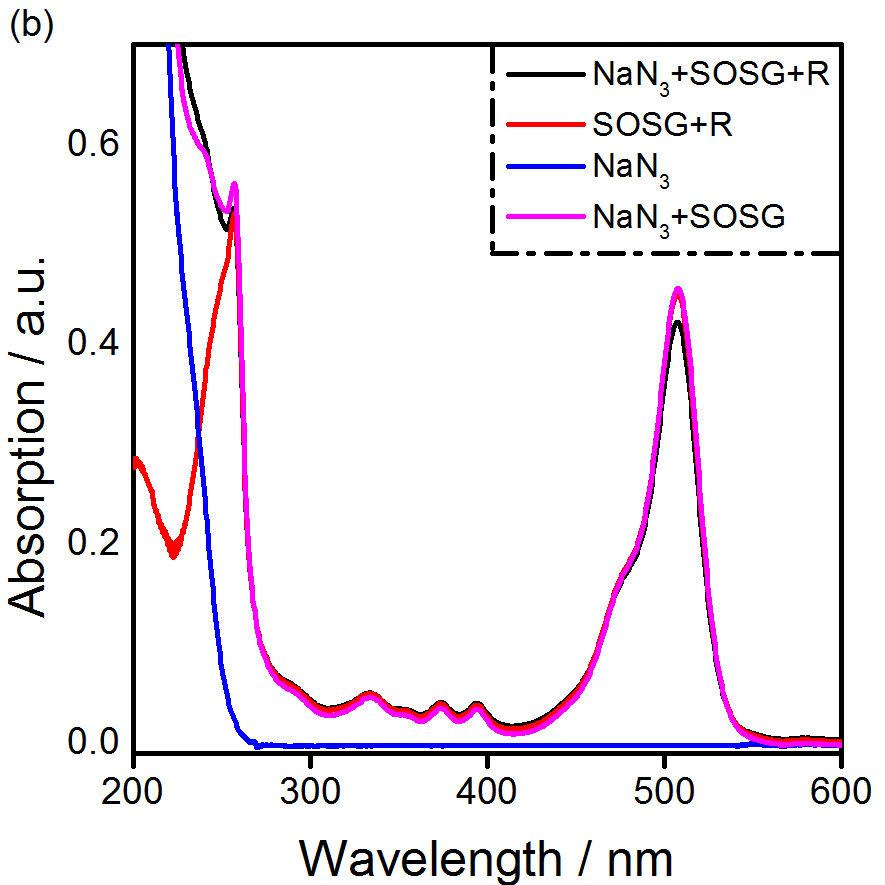


Figure S5. (a) UV-vis spectrum of SOSG solutions (5 µM) with the addition of ethanol(0.85 M) in the absence and presence of ionizing gamma radiation; (b) UV-vis spectrum of SOSG solutions with the addition of NaN_3_ (1 mM)in the presence and absence of ionizing gamma radiation (‘R’ stands for a radiation exposure of 40 Gy from a ^60^Co source.).


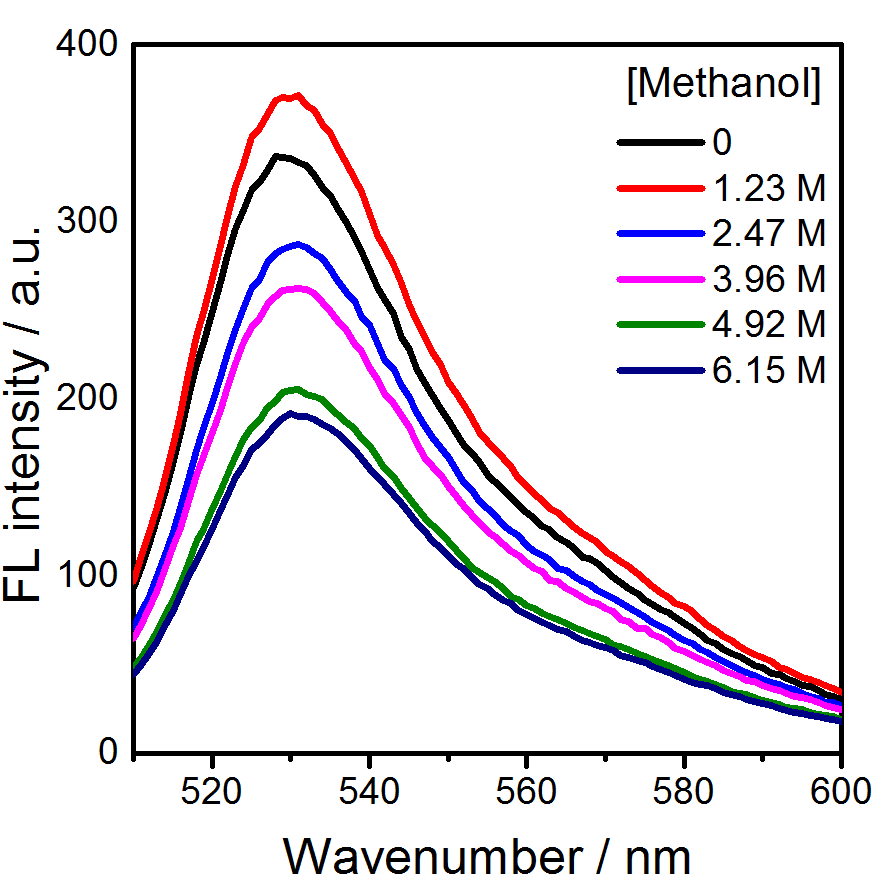


Figure S6. Fluorescence spectra (λ_ex_= 504 nm) of SOSG solutions in the presence of different concentrations of methanol and when exposed to a radiation dose of 20 Gy delivered by a ^60^Co source.


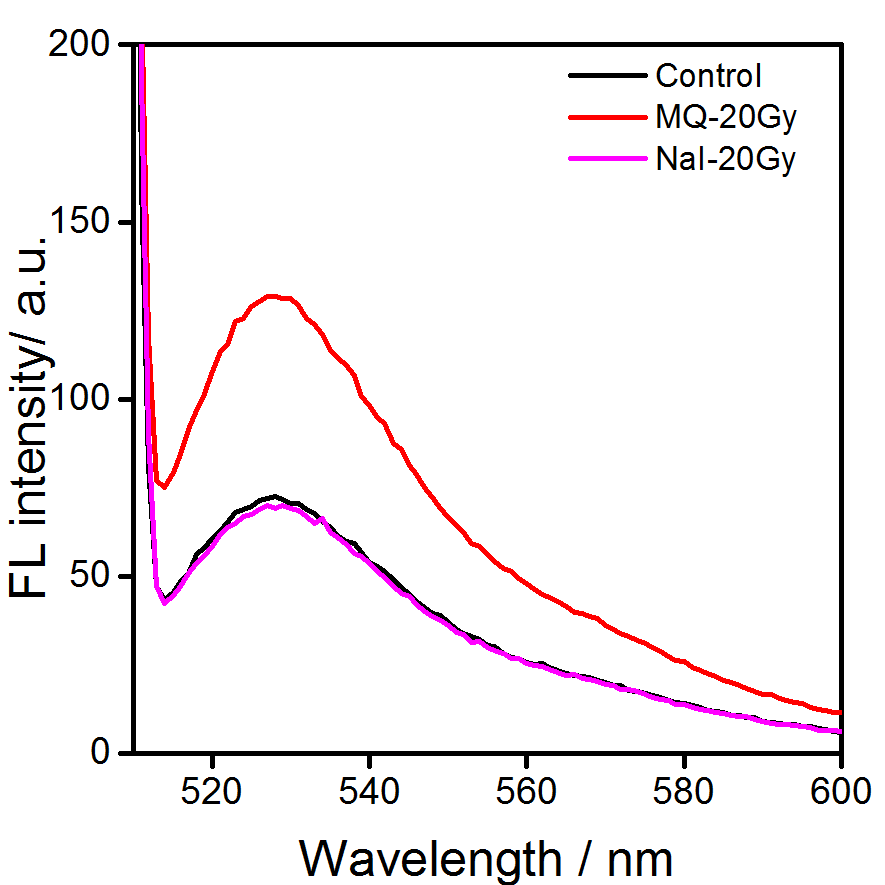


Figure S7. Fluorescence spectra (λ_ex_= 504 nm) of SOSG solutions in the presence of NaI (50 mM) and when exposed to a radiation dose of 20 Gy delivered by a ^60^Co source.


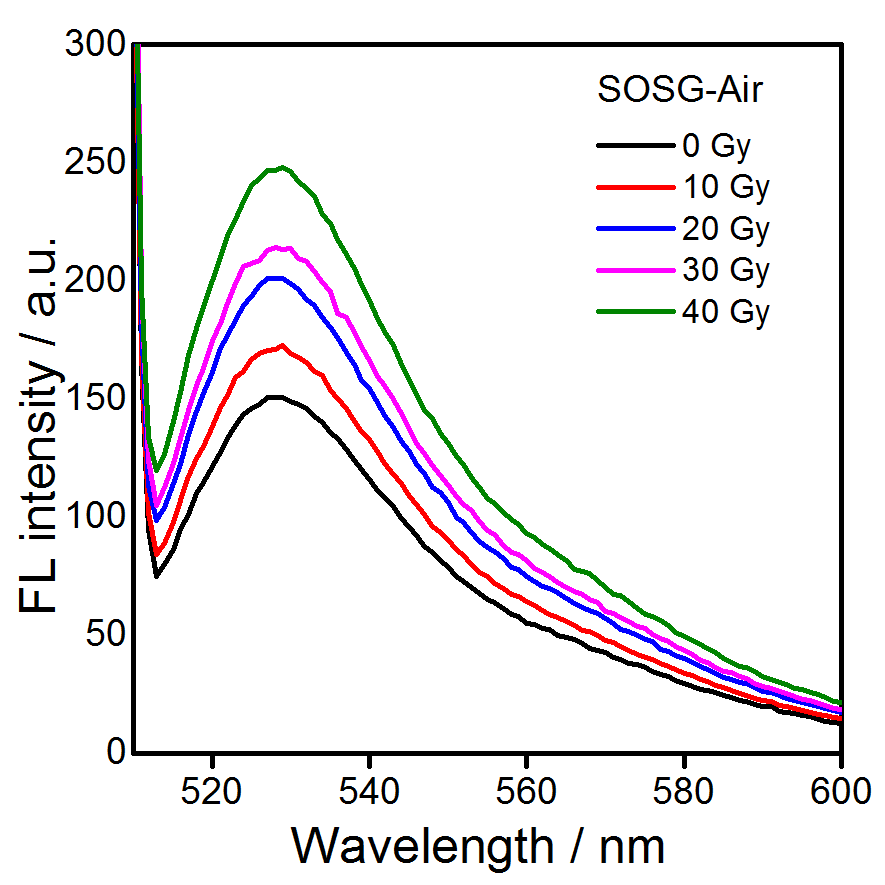


Figure S8. Fluorescence spectra (λ_ex_= 504 nm) of SOSG solutions (5 µM) (e) saturated with N_2_ for varied radiation doses delivered by a ^60^Co source


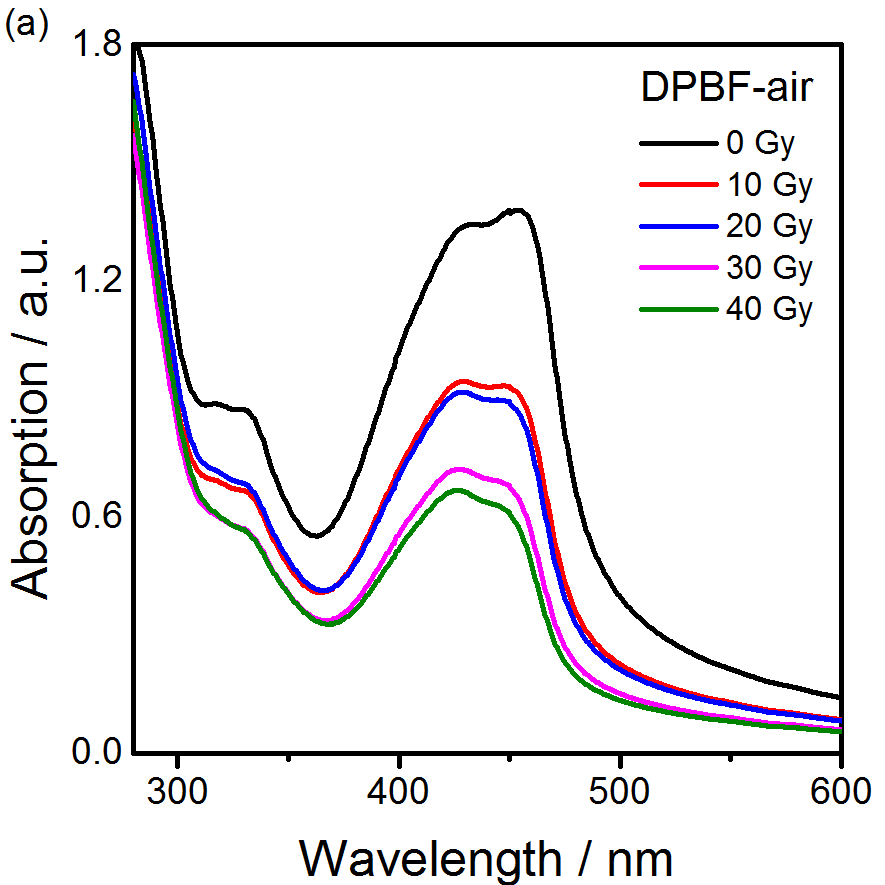

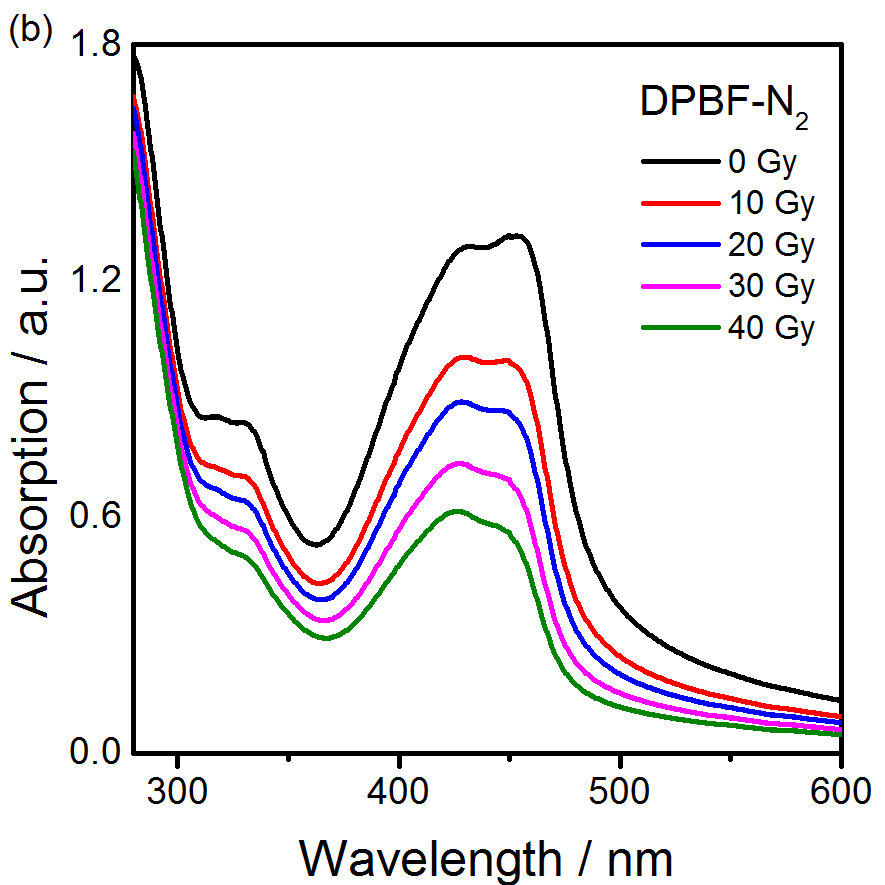

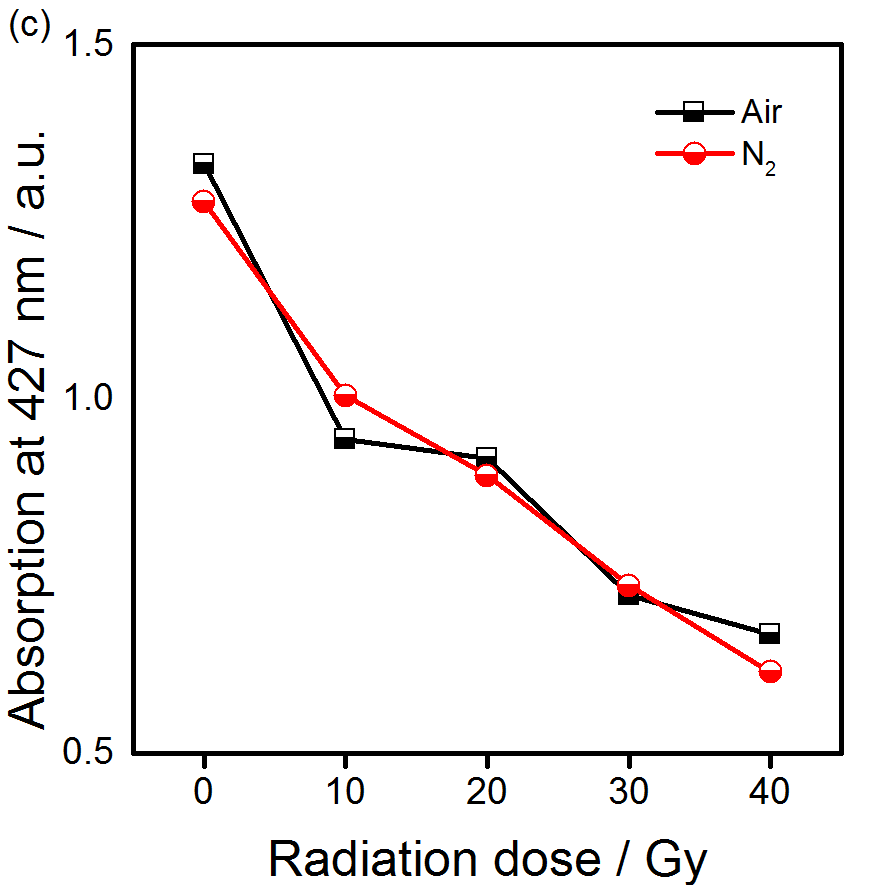


Figure S9. UV-vis spectra for DPBF solutions (100 µM) (a) saturated with air and (b) saturated with N_2_ for varied radiation doses delivered by a ^60^Co source; (c) The comparison of the intensity at 427nm between air-saturated SOSG solutions and N_2_-saturated SOSG solutions after exposure to different gamma-ray doses.


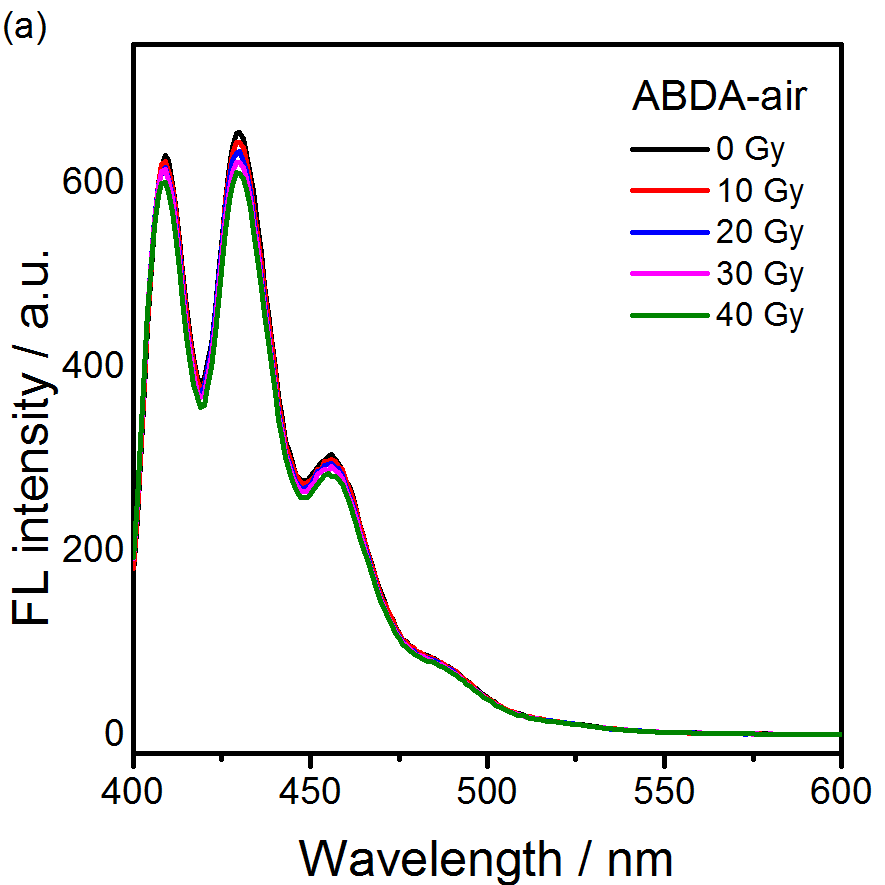

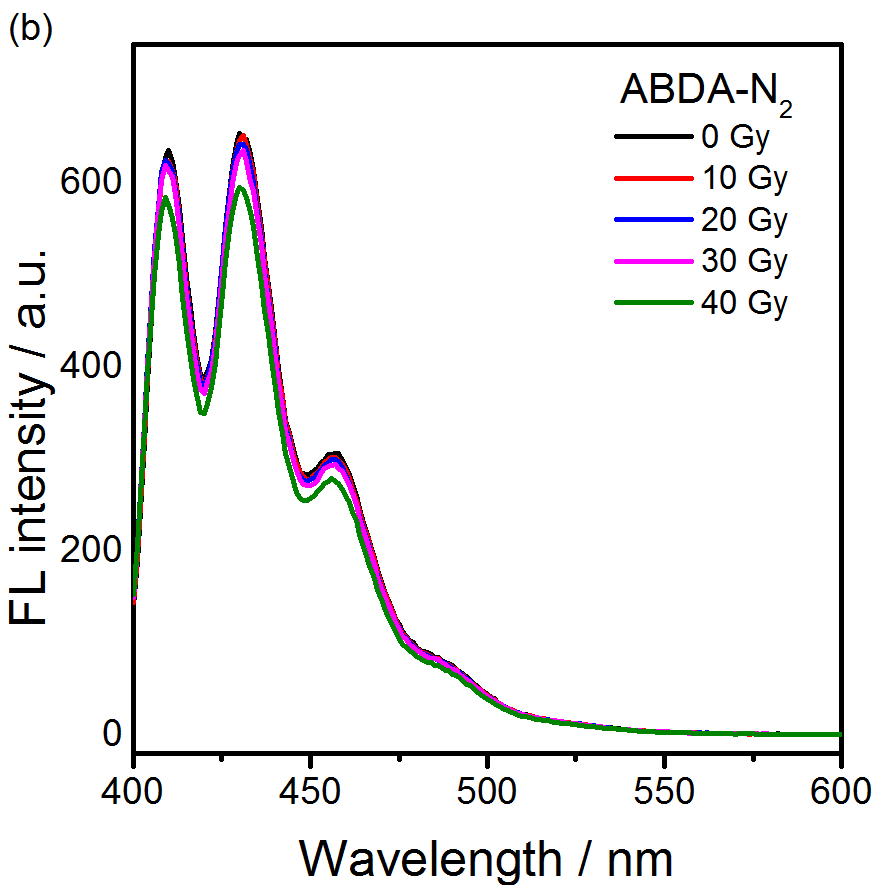

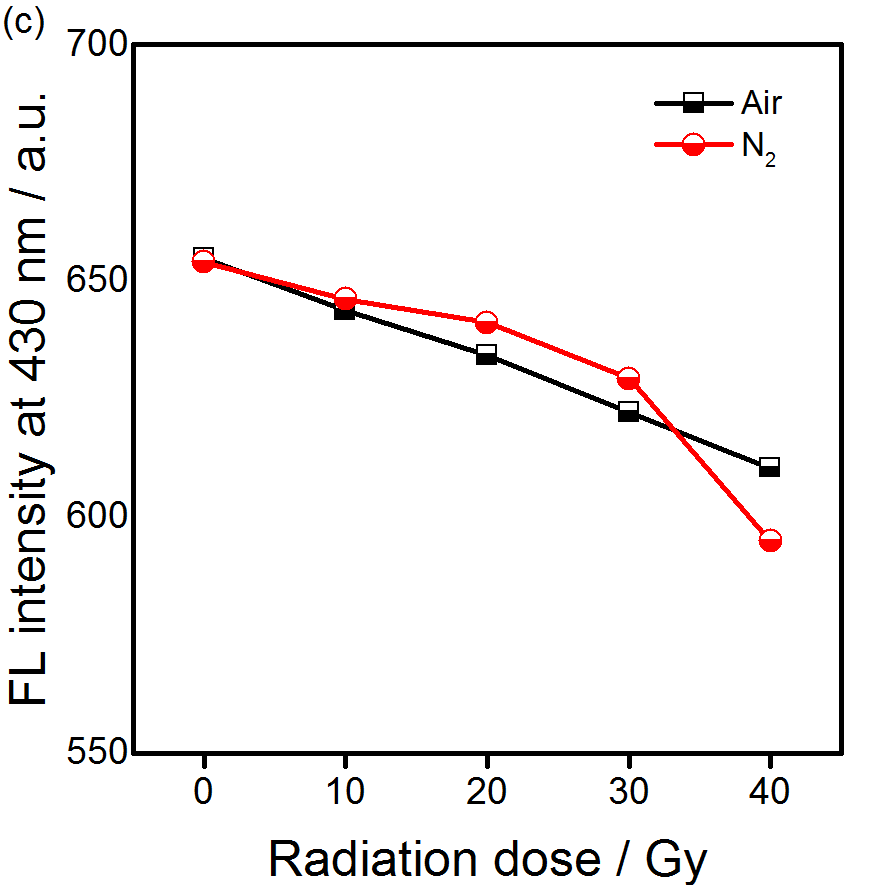


Figure S10. Fluorescence spectra ( λ_ex_= 380 nm) of ABDA solutions (50 µM) (a) saturated with air and (b) saturated with N_2_ for varied radiation doses delivered by a ^60^Co source; (c) The comparison for the FL intensity at 430 nm between air-saturated SOSG solutions and N_2_-saturated SOSG solutions after exposure to different gamma-ray doses.


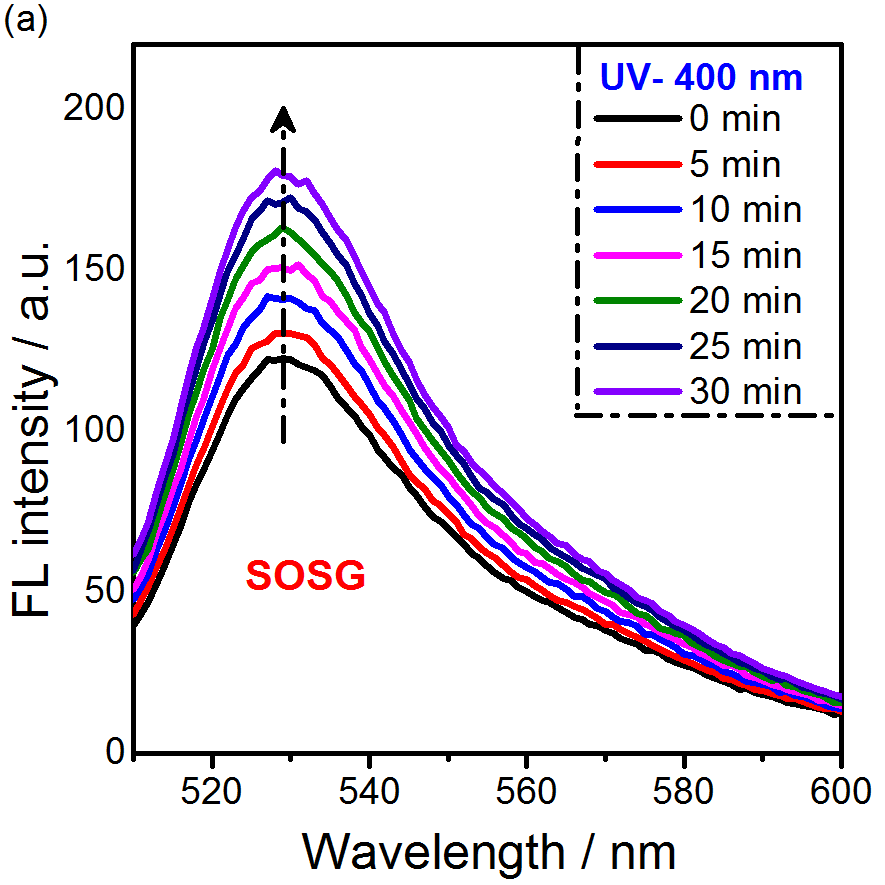

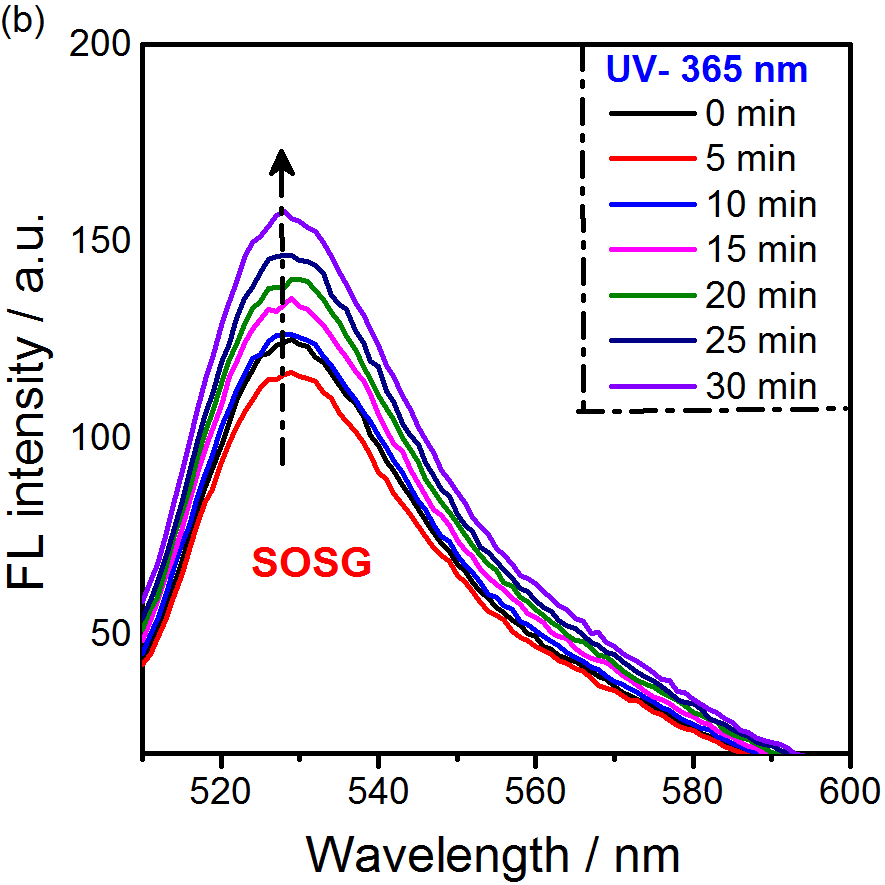


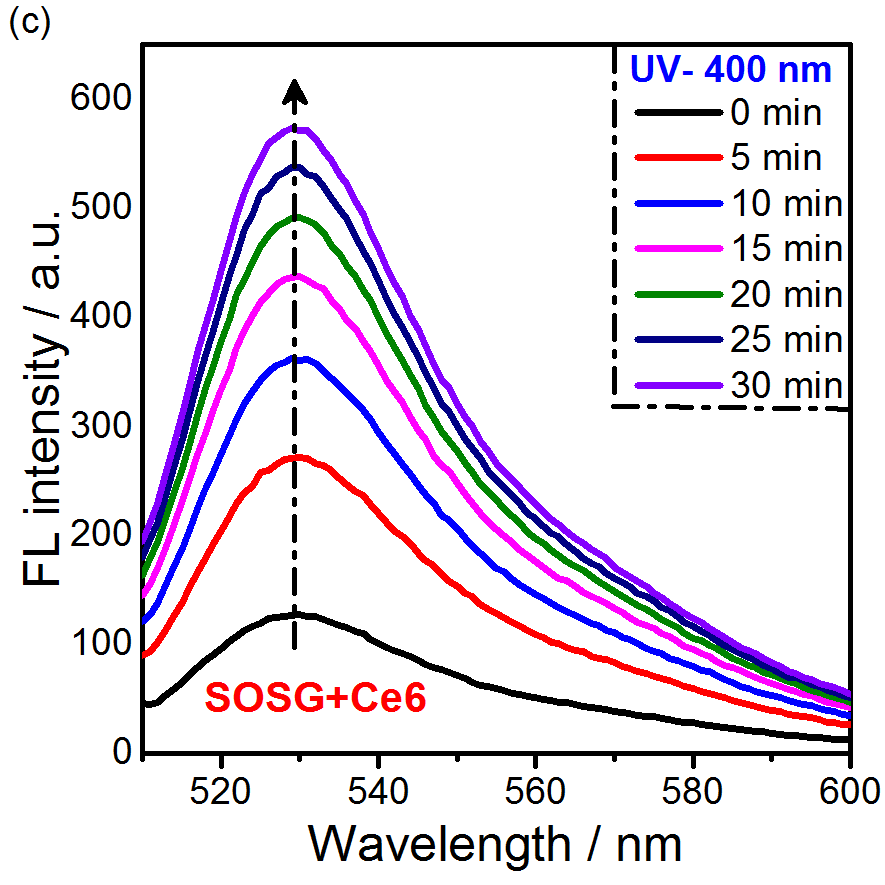

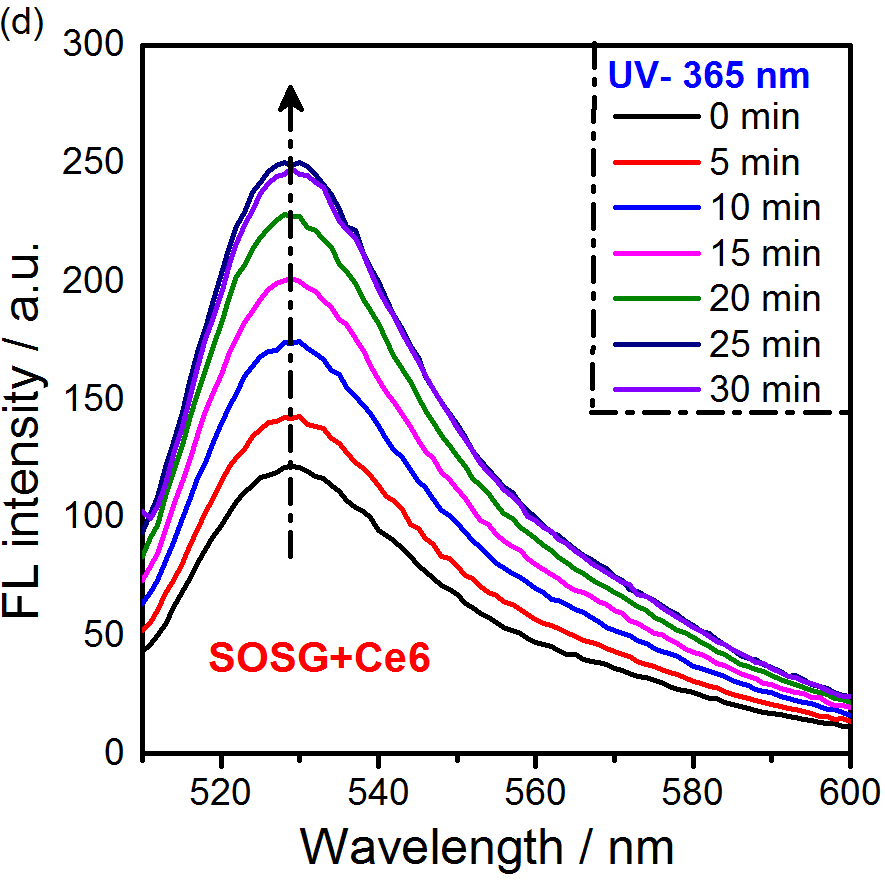


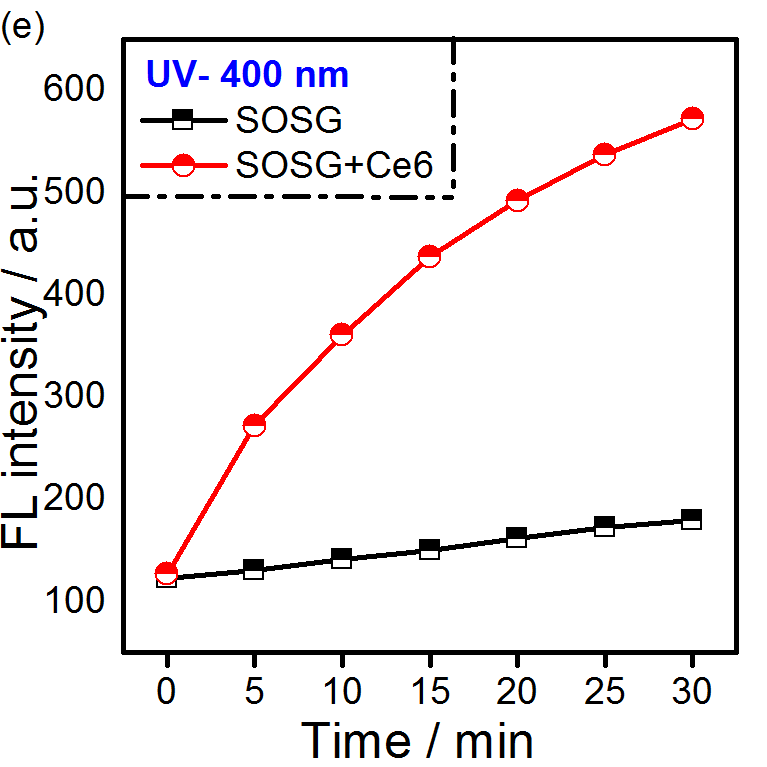

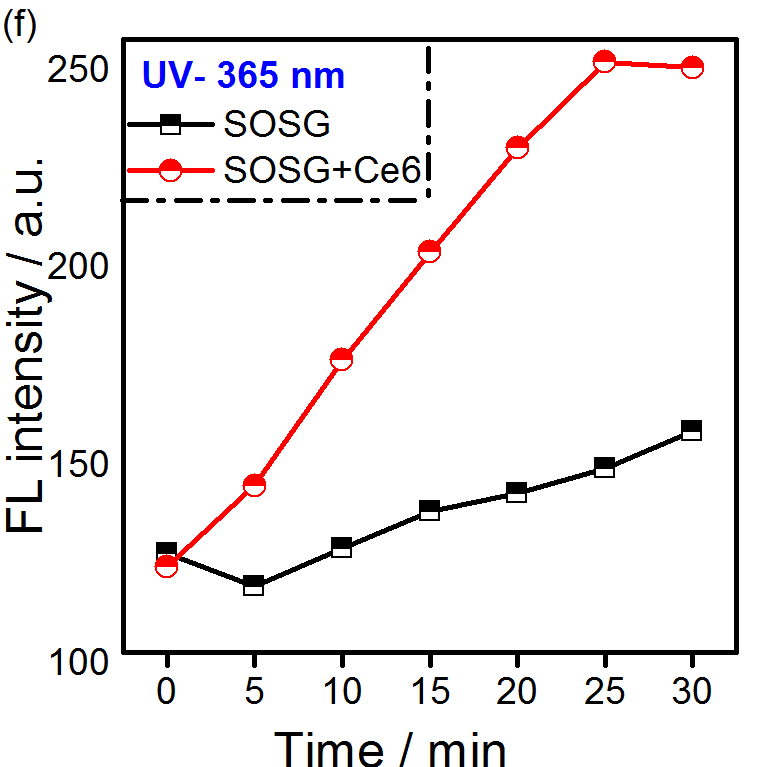


Figure S11. Fluorescence spectra (λ_ex_= 504 nm) of SOSG solutions (5 µM) after irradiation with UV light at (a) 400 nm and (b) 365 nm; Fluorescence spectra of SOSG (5 µM)+Ce6 (5 µM) mixtures after irradiation with UV light at (c) 400 nm and (d) 365 nm; Comparison of the fluorescence intensity at ~530 nm between pure SOSG solutions (5 µM) and SOSG solutions (5 µM) containing Ce6 (5 µM) after irradiation with UV light at (e) 400 nm and (d) 365 nm.

Figure S11 show that both solutions, i.e. SOSG and SOSG+Ce6 have increased fluorescence intensity with increased UV irradiation time, which means that SOSG can act as a photosensitizer and produce singlet oxygen leading to increased fluorescence. The solution containing photosensitizer Ce6 renders much higher FL intensity than that for the pure SOSG system.

References

1. X. Ragas, A. Jimenez-Banzo, D. Sanchez-Garcia, X. Batllori & S. Nonell, Singlet oxygen photosensitisation by the fluorescent probe Singlet Oxygen Sensor Green®. *Chem*. *Commun*., 2920-2922 (2009).
2. Chao Zhou, Naiyun Gao, Yang Deng, Wenhai Chu, Wenlei Rong & Shengdong Zhou, Factors affecting ultraviolet irradiation/hydrogen peroxide (UV/H_2_O_2_) degradation of mixed N-nitrosamines in water. *J. Hazard. Mater*. **231-232**, 43-48 (2012).
